# Whole-exome sequencing identified a novel heterozygous variant in *UBAP2L* in a Chinese family with neurodevelopmental disorder characterized by impaired language, behavioral abnormalities, and dysmorphic facies

**DOI:** 10.3389/fgene.2024.1503048

**Published:** 2024-12-10

**Authors:** Qi Yang, Qiang Zhang, Xunzhao Zhou, Juntan Feng, Shujie Zhang, Li Lin, Shang Yi, Zailong Qin, Jingsi Luo

**Affiliations:** ^1^ Guangxi Key Laboratory of Birth Defects Research and Prevention, Guangxi Key Laboratory of Reproductive Health and Birth Defects Prevention, Maternal and Child Health Hospital of Guangxi Zhuang Autonomous Region, Nanning, China; ^2^ Department of Genetic and Metabolic Central Laboratory, Maternal and Child Health Hospital of Guangxi Zhuang Autonomous Region, Nanning, China; ^3^ Department of Pediatric Neurology, Maternal and Child Health Hospital of Guangxi Zhuang Autonomous Region, Guangxi Clinical Research Center for Pediatric Diseases, Nanning, China; ^4^ Guangxi Clinical Research Center for Pediatric Diseases, Maternal and Child Health Hospital of Guangxi Zhuang Autonomous Region, Nanning, China

**Keywords:** UBAP2L-deficiency syndrome, *UBAP2L*, intellectual disability, developmental delay, Chinese

## Abstract

UBAP2L-deficiency syndrome, also known as neurodevelopmental disorder with impaired language, behavioral abnormalities, and dysmorphic facies (NEDLBF, OMIM 620494), is an extremely rare autosomal dominant disorder. This condition is caused by heterozygous variant in the *UBAP2L* gene (NM_014847.4, MIM 616472), which encodes the ubiquitin-associated protein 2-like protein involved in the formation of stress granules (SGs). To date, only one report has documented 12 loss-of-function variants in *UBAP2L*, all of which were identified as *de novo* variants. In our study, we recruited a Chinese family with two patients exhibiting intellectual disability and seizures. Whole-exome sequencing was performed on the proband, revealing a novel heterozygous frameshift variant, *UBAP2L* (NM_014847.4):c.2453_2454del (p.Tyr818Trpfs*3). The variant was inherited from the affected mother, and confirmed in the proband and his parents by Sanger sequencing. This is the first familial report of a deleterious *UBAP2L* variant. The proband in this family presented a clinical phenotype similar to NEDLBF, which includes intellectual disability, developmental delay, speech delay, facial dysmorphism, seizures, and behavioral abnormalities. The affected mother presented only mild intellectual disability and mild language impairment. By clinical evaluation of our patients and previously reported cases with *UBAP2L* variants, we propose that intellectual disability, developmental delay (particularly in speech), infants’ feeding difficulties, behavioural abnormalities and seizures are the main clinical features of NEDLBF patients. Our study expands the genetic and phenotypic spectrum associated with NEDLBF.

## Introduction

**FIGURE 1:**
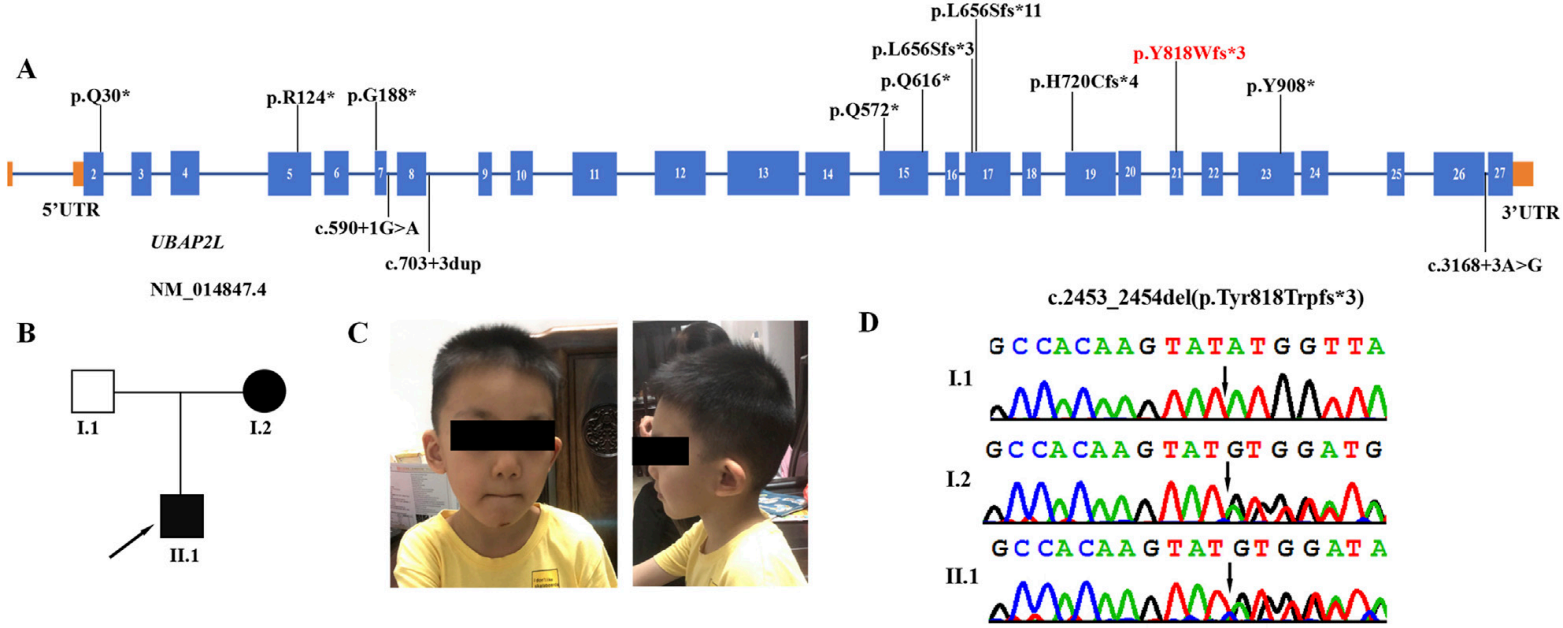
Clinical and genetic features. **(A)** The spectrum of *UBAP2L* pathogenic variants. The red variant is the novel variant identified in this study. **(B)** Family pedigree showing that both the mother and the proband are affected. **(C)** Facial appearance of the proband (II-1) at the age of 4 years old, showing cupped ear, long face and short nose. **(D)** DNA sequence chromatograms from Sanger sequencing of *UBAP2L*, showing a heterozygous frameshift variant *UBAP2L* (NM_014847.4: c.2453_2454del (p.Tyr818Trpfs*3)) in the proband. Sanger sequencing further revealed that his affected mother was heterozygous for the same variant and that his father was normal.

UBAP2L-deficiency syndrome, also known as neurodevelopmental disorder with impaired language, behavioral abnormalities, and dysmorphic facies (NEDLBF, OMIM 620494), is caused by heterozygous loss-of-function variants in the *UBAP2L* gene, located at 1q21.3 (Jia et al., 2022). This gene contains 27 exons and encodes the ubiquitin-associated protein 2-like protein, which is involved in the formation of stress granules (SGs). SGs play a crucial role in cell survival under stress conditions and have been implicated in the pathogenesis of cancer, neurodegeneration, inflammatory diseases, and viral infections (Panas et al., 2016; Buchan et al., 2008; Arimoto et al., 2008; Takahara and Maeda, 2012). Moreover, UBAP2L is involved in the ubiquitination and degradation of RNA polymerase II (RNAPII) by recruiting Cullin-based ubiquitin complexes (Herlihy et al., 2022). Additionally, UBAP2L is a spindle-associated protein that plays a significant role in cellular mitosis by regulating the activity of the kinase PLK1 (Guerber et al., 2023). It has been identified as an oncogene associated with various cancers, including glioma, prostate cancer, hepatocellular carcinoma (HCC), breast cancer, and colorectal cancer (Li and Huang, 2014; Zhao et al., 2015; Ye et al., 2017; He et al., 2018; Chai et al., 2016). Furthermore, Lingerer, the *Drosophila* homolog of the human *UBAP2L* gene, regulates the proliferation of growing tissues by modulating the JAK/STAT signalling pathway (Baumgartner et al., 2013). A recent study found that variants in the *UBAP2L* gene were associated with developmental delay (DD), speech delay, mild-to-severe intellectual disability (ID), feeding difficulties in infants, seizures, motor delay, various behavioral abnormalities, hypotonia, skeletal anomalies, facial dysmorphism, and other variable clinical features (Jia et al., 2022) (Figure 1A).

Here we report the first familial case of NEDLBF syndrome in a Chinese family with a novel pathogenic variant of *UBAP2L* (NM_014847.4):c.2453_2454del (p.Tyr818Trpfs*3) (Figure 1B). The mother carries this novel variant and passed it on to the proband. We provide a detailed description of the clinical phenotypes observed in both the proband and his mother. This report expands the spectrum of variants in the *UBAP2L* gene and provides additional molecular and clinical information insights to enhance our understanding of UBAP2L-deficiency syndrome.

## Material and methods

### Patients and ethics approval

The proband was initially referred to the Paediatric Endocrine Clinic of the Guangxi Maternal and Child Health Hospital due to behavioral issues and seizures. Written informed consents for publishing data and images were obtained from participants and the parents of the participant under the age of 16. The study was approved by the the Medical Ethics Committee of the Maternal and Child Health Hospital of Guangxi Autonomous Region. The patient was admitted following standard admission protocols, which included a comprehensive medical history, laboratory tests, physical examination, abdominal ultrasound, neurological examination, electroencephalogram, magnetic resonance imaging (MRI) of the brain, audiological examination, routine ophthalmological examination, and genetic testing.

### Whole exome sequencing and sanger sequencing

We collected 5 mL peripheral blood samples from the proband and his parents for whole-exome sequencing. Genomic DNA was isolated from peripheral blood samples using the Lab-Aid DNA kit (Zeshan Biotechnology Co., Ltd., Xiamen, China), and its concentration and purity were assessed using a NanoDrop 1000 spectrophotometer (Thermo Scientific). Sequencing libraries were prepared using Agilent SureSelect Human Exon V6 kits (Agilent Technologies, Santa Clara, CA, United States) according to standard Illumina protocols and then sequenced with 100 bp paired-end reversible terminator technology on the Illumina Hiseq2000 platform at Huada Gene Technology Co. Ltd. (Shenzhen, China). Burrows-Wheeler Aligner software version 0.6.2 was used to map the clean reads to the the human genome assembly GRCh37. Annotation and classification of variants were performed using TGex software (LifeMap Sciences, Alameda, CA, United States). The variant analysis included those variants with a minor allele frequency of less than 1% in public databases (e.g., 1000Genomes Project, Genome Aggregation Database, Exome Sequencing Project, and ExAC) and in our in-house databases. The deleterious effect of the selected variants was evaluated using *in silico* prediction tools such as Variant Taster, CADD, SIFT, and PolyPhen2. Final interpretation and categorization of variants was performed using criteria developed by the American College of Medical Genetics (ACMG)/Association for Molecular Pathology (AMP) (Richards et al., 2015).

Sanger sequencing was used to validate the identified variants in the proband and his family members. Sanger primers flanking the candidate variant alleles were custom designed using Primer 3 software (https://bioinfo.ut.ee/primer3-0.4.0/primer3/) (forward primer: 5′-TCC​GTG​TCC​TGT​TCT​AAC​CC-3′ and reverse primer: 5′- GCT​GGG​ATG​GGA​AAG​AGC​TA-3′). PCR amplification was performed using Takara PrimeSTAR Max DNA Polymerase (Takara Biotechnology Co., Ltd., Dalian, China) under the following conditions:one cycle of initial denaturation at 95°C for 5 min; 35 cycles consisting of 30 s at 95°C, 30 s at 60°C, and 30 s at 72°C; followed by a final extension at 72°C for 5 min. The purified PCR fragments were then sequenced on an ABI 3500DX DNA analyzer (Applied Biosystems; Thermo FisherScientific, Inc.) using the Big Dye Terminator Cycle sequencing reaction kit (Perkin-Elmer, Applied Biosystems Division, Foster City, CA).

## Results

### Clinical phenotype

The proband was a Chinese boy and the first child of non-consanguineous parents. He was born prematurely at 34 weeks of gestation with a birth weight of 2,180 g (49.4^th^ percentile), a birth length of 41.5 cm (50th percentile), a head circumference of 31 cm (48th percentile) and Apgar scores of 9, 9, and 10 at 1, 5, and 10 min, respectively. The proband was first admitted to Paediatric Department of the Guangxi Zhuang Autonomous Region Maternal and Child Health Hospital at the age of 4 years due to behavioral issues and seizure. He experienced mild developmental delay, achieving head control at 5 months, crawling at 10 months, and walking independently at 19 months. Speech development was delayed, with single words spoken at 20 months and complete sentences at 2 years and 4 months. Mild autistic traits were noted, including poor eye contact, repetitive behaviors, avoidance of group play, and unusual fixations and obsessions. At age 4, the Gesell Developmental Diagnostic Scale was used to assess his Developmental Quotient (DQ, DQ < 70 as low score): gross motor 80, fine motor 76, adaptive 58, language 57, and personal-social 56. A physical examination revealed that his height (102 cm, 25th percentile) and weight (15 kg, 25th percentile) were within normal ranges. Mild dysmorphic features included cupped ears, a long face, and a short nose (Figure 1C). The proband experienced his first seizure at 6 months of age during a fever, characterized as absence seizures occurring twice daily, each lasting about 5 s. Sleep electroencephalogram results showed spike, spike-slow, and polyspike-slow wave distributions in the right middle temporal region. Over the following years, he experienced several episodes of absence seizures, each coinciding with a very high fever (over 39°C) at onset. His seizures were controlled with valproate (VPA) treatment. The initial brain MRI at age 4 was normal.

Family history revealed that the proband’s mother experienced several absence seizures with high fever as a child which ceased after she began taking valproic acid (VPA). The mother, aged 34, has a normal height of 158 cm (30th percentile), and there was no sign of short stature during her childhood. She exhibits mild intellectual disability (IQ = 68) and mild language impairment. No behavioral abnormalities or dysmorphic features were observed. Unfortunately, the proband’s mother was unable to return for an EEG or brain MRI.

### Genetic analysis

To determine the genetic etiology of the disease, whole-exome sequencing was performed on the proband. A total of 7.1 Gb of data was generated, with 99.4% of the target region covered and 99.0% of the targets covered more than 20X. There were 24,356 single nucleotide variants (SNVs) or insertion/deletion (indel) variants identified in the coding region and splice site (within 10 bp from the splice junction). After filtering out synonymous variants and those with a minor allele frequency (MAF) greater than 1% in public databases (e.g., 1000 Genomes Project, Genome Aggregation Database, Exome Sequencing Project and ExAC) as well as our in-house databases, a total of 758 variants were remained. Using TGex software (LifeMap Sciences, United States), 9 candidate variants in 9 genes (*UBAP2L*, *CUX2*, *CACNA1I*, *SAMD12*, *HERC2*, *IQSEC1*, *NFASC*, *RERE*, *PLAA*) were mapped to known phenotypes including seizures, intellectual disability, developmental delay, motor delay, language delay and autistic behaviour. The variants in the *HERC2*, *IQSEC1*, *NFASC* and *PLAA* genes were heterozygous (Supplementary Table S1). The disorders resulting from these genetic variants are autosomal recessive and have therefore been ruled out. The variants in the *CUX2, CACNA1I*, *RERE* and *SAMD12* genes were transmitted from the unaffected father (Supplementary Table S1). Subsequently, a heterozygous frameshift variant located in exon 21 of *UBAP2L* (c.2453_2454del (p.Tyr818Trpfs*3)) was identified in the proband. Further Sanger sequencing determined that the variant was inherited from the probands’ mother. The frameshift variant was classified as likely pathogenic according to the ACMG/AMP guidelines. No pathogenic or likely pathogenic CNVs were detected in the WES data of the patient using the XHMM software and the depth of coverage methods.

## Discussion

UBAP2L, also known as NICE-4, is a highly conserved protein involved in various cellular biological processes, including spindle assembly, chromosome segregation, and regulation of cell proliferation and division (Guerber et al., 2023; He et al., 2018; Maeda et al., 2016). In a recent study by Jia et al., in 2022, loss-of-function variants in *UBAP2L* were associated with neurodevelopmental disorders (Jia et al., 2022). Their research demonstrated that *Ubap2l* knockout (KO) mice exhibited a lethal phenotype in most embryos, with a minority (2.6%) of surviving mice displaying significantly reduced size. Furthermore, heterozygous defective mice exhibited diminished preference for social novelty, cognitive deficits, and anxiety-like behavior, indicating a role for UBAP2L dysfunction in the neurodevelopmental deficits observed in affected individuals. In this report, we identified a novel heterozygous frameshift variant (c.2453_2454del (p.Tyr818Trpfs*3)) in the *UBAP2L* gene of a patient with a neurodevelopmental disorder (NDD) (Figure 1D). Sanger sequencing revealed that the affected mother was heterozygous for the same variant, while the unaffected father was not. This variant, located in exon 21 of the *UBAP2L* gene, introduces a premature termination codon that may activate the nonsense-mediated mRNA decay (NMD) pathway, resulting in a significant reduction in UBAP2L mRNA expression levels and subsequent loss of function. The variant is novel and is not present in the Human Gene variant Database (http://www. hgmd. cf.ac.uk/ac/), HPSD (http://liweilab.genetics. ac. cn/HPSD/), dbSNP (http://www.ncbi.nlm.nih.gov/SNP/), ExAC and gnomAD (https://gnomad.broad institute. org/). Based on the ACMG/AMP classification guidelines, it is classified as a likely pathogenic variant (PVS1+PM2_supporting). This finding confirms that *UBAP2L* variants are likely responsible for the neurodevelopmental abnormalities in this family.

To date, only 14 affected individuals (including our patient) worldwide have been reported with *UBAP2L* variants (Jia et al., 2022). All identified variants (five frameshifts, three splicing, and six nonsense) generate null alleles (Figure 1D). The reported *UBAP2L* variants and the available clinical phenotypes for all cases are summarized in Table 1. Phenotypic analyses of affected individuals revealed significant heterogeneity in the characteristics observed in patients with *UBAP2L* loss-of-function variants; however, certain common characteristics (>50% of cases) could be identified. All cases exhibited neurodevelopmental abnormalities across multiple areas, ranging from mild to severe. Facial dysmorphic features were observed in almost all patients (10/11), but there was no consistency-some patients having only mild synophrys or epicanthus, while others having a variety of severe facial deformities. The speech domain appeared particularly vulnerable, with all patients (13/13) showing speech delays, and about half of them exhibiting marked delays, suggesting that language disorders are a typical symptom of UBAP2L deficiency syndrome. Intellectual disability was observed in 10 out of 12 patients, and motor delay was noted in 9 out of 13. Notably, the severity of these conditions was relatively mild, with only one patient exhibiting moderate to severe intellectual disability. Our patients also had mild intellectual disability and motor delay. Behavioral problems were reported in 10 of the 14 patients, including anxiety, ADHD, repetitive behaviors, obsessive behaviors, aggressive behaviors, self-injurious behaviors, emotional issues, and trichotillomania. Notably, the proband in this study exhibited autistic behaviors, such as repetitive actions, avoidance of group play, and unusual fixations and obsessions, whereas his mother did not display these issues. Multiple types of seizures were observed in most patients (9/14), with febrile seizures being the most common type. In the present study, both the proband and his mother had absence seizures during fever, which were controlled after treatment with valproic acid (VPA). Electroencephalography (EEG) abnormality were observed in four patients (4/7), including our proband. Most patients (8/12) experienced feeding difficulties in infancy, which improved with age. The proband in this study also had feeding difficulties during infancy. Skeletal and limb abnormalities were observed in six patients (6/13), including mild strephenopodia, clinodactyly of the V finger, lower limb skeletal anomalies (metatarsal adducts, femoral anteversion, tibial torsion), brachydactyly, joint stiffness (hands, elbows, knees, and feet), symphalangism of the thumb, scoliosis, kyphosis, nail hypoplasia, pointing finger joint contracture, broad halluces, proximally set thumbs, misalignment of the elbows and wrists, and prehensile/elongated halluces with varus deformity. Another area of concern is vision problems; more than half of the patients experienced issues in this domain, and early correction and treatment could benefit these children. Additionally, variable manifestations were observed among these patients. Several had failure to thrive (4/9), short stature (4/11), and microcephaly (4/11). Three patients had gastrointestinal problems, two had brain abnormalities, and two experienced sleep issues. Other dysmorphic features, such as hypertrichosis, café au lait spots, chronic bronchitis, recurrent urinary tract infections, asthma, and cutis marmorata, were also noted. In contrast, our patients did not have these variable dysmorphic features, including skeletal abnormalities, visual problems, failure to thrive, short stature, microcephaly, gastrointestinal problems, brain abnormalities, and other rare dysmorphic features.

**TABLE 1:** Summary of the genetic and clinical features of the patients with UBAP2L-deficiency syndrome.

	Jia et al. (2022)												Our patients		Total
	P1	P2	P3	P4	P5	P6	P7	P8	P9	P10	P11	P12	P13	P14	N = 14
Variants in *UBAP2L* (NM_014847.4)	c.88C>T (p.Q30*)	c.370C>T (p.R124*)	c.562G>T (p.G188*)	c.590 + 1G>A (p.G182Efs*78)	c.703+3dup (p.T198Cfs*12)	c.1714C>T (p.Q572*)	c.1846C>T (p.Q616*)	c.1964dup (p.L656Sfs*3)	c.1965del (p.L656Sfs*11)	c.2158_2165del (p.H720Cfs*4)	c.2724C>A (p.Y908*)	c.3168 + 3A>G (p.Q991_Q1056del)	c.2453_2454del (p. Y818Wfs*3)	c.2453_2454del (p. Y818Wfs*3)	Frameshift = 5 Splicing = 3 nonsense = 6
Inheritance	*de novo*	*de novo*	*de novo*	*de novo*	*de novo*	*de novo*	*de novo*	*de novo*	*de novo*	*de novo*	*de novo*	*de novo*	Maternal	NA	
Affected exon/intron	2	5	7	7	8	15	15	17	17	19	23	26	21	21	
Gender	male	male	female	female	female	female	male	male	male	male	female	female	male	female	Male = 7; female = 7
Age at last examination	3y9m	9y	NA	7y	15y8m	9y	15y9m	15y4m	13y9m	4y2m	4y6m	6y	4y	30y	
Feeding difficulties (HP:0011968)	+	NA	+	+	—	+	—	+	+	+	—	+	—	NA	8/12
Failure to thrive (HP:0001508)	+	NA	NA	+	—	NA	—	NA	+	—	—	+	—	NA	4/9
Intellectual disability (HP:0001249)	moderate to severe	mild	NA	mild	mild	mild	—	—	mild	+	+	NA	+	+	10/12
Short stature (HP:0004322)	+	NA	NA	+	—	—	—	—	—	+	—	+	—	—	4/12
Microcephaly (HP:0000252)	+	NA	NA	—	+	-	-	-	—	+	—	+	—	—	4/12
Behavioral abnormality (HP:0000708)	—	+	+	+	+	+	+	+	—	+		+	+	—	10/14
Delayed speech and language development (HP:0000750)	+	NA	+	+	+	+	+	+	+	+	+	+	+	+	13/13
Motor delay (HP:0001270)	+	—	+	—	+	+	+	—	+	+	—	+	+	NA	9/13
Seizure (HP:0001250)	—	—	Febrile seizures	—	At age of 1 year: tonic-clonic seizures during fever aNA without fever. The seizures mainly affected one hemisoma. At age of 3 years she experienced absence seizures	+	—	—	Epileptic seizures only one time	History of one febrile seizure brought on by 104° fever	Febrile seizures-first seizure at 18 months	Convulsive seizure with flu,Symptomatic generalized epilepsy: non-convulsive absence seizures	Absence seizures during fever	+	9/14
EEG abnormality (HP:0002353)	NA	NA	NA	—	+	NA	—	NA	+	NA	—	+	+	+	5/8
Brain imaging abnormality (HP:0410263)	—	—	NA	—	Malacic lesion at white mater of right occipital horn, ex-vacuo dilation of right occipital horn	NA	—	—	Mild vermis hypoplasia and a thin corpus callosum; No structural abnormalities	—	—	—	—	NA	2/10
Sleep abnormality (HP:0002360)	—	—	NA	—	—	NA	Sleep apnea diagnosed	Daytime sleepiness	—	—	-	NA	—	NA	2/10
Functional abnormality of the gastrointestinal tract (HP:0012719)	—	—	NA	Gastroesophageal Reflux	—	NA	Hyperphagia, prior history of abdominal with diarrhea alternating with constipation	—	—	Acid reflux issues were noted shortly after birth	—	NA	—	—	3/11
Abnormality of the skeletal system (HP:0000924)	Mild strephenopodia; Digital pudds; Clinodactyly V finger	—	Lower limb skeletal anomalies (metatarsal adducts, femoral anteversion, tibia torsion)	Brachydactyly; joint stiffness (hands, elbows, knees, and feet); symphalangism of thumb	—	—	Scoliosis and kyphosis	NA	—	Nails hypoplasia; Pointing finger joint contracture; Broad halluces	—	Proximately set thumbs; misalignment of the elbows and wrists, Prehensile/elongated halluces with R varus deformity; brachydactyly of the toes	—	—	6/13
Visual impairment (HP:0000505)	Congenital abnormal eye development (nystagmus, leukoma, esotropia	—	NA	Mild hypermetropic astigmatism	—	—	Hpermetropia, blinks very frequently	Corrected	Myopia	Exotropia in right eye	—	NA	—	—	6/12
Muscular hypotonia (HP:0001252)	—	—	+	—	NA	+	NA	NA	+	NA	—	+	—	—	4/10
Facial dysmorphism	Round face; Asymmetrical palpebral fissure; Cup ears; Low front hairline; Deviated mouth; Deep and prominent concha	NA	NA	Mild synophrys; Upslanting palpebral fissures; Deep aNA prominent concha	Mild synophrys	Large front horizontal eyelashes; Hypertelorism; Broad nasal bridge; Ears small helix (scrumpled)	Long face with facial asymmetry; Palpebral fissures are straight (interpupillary distance is 53 mm); Tubular nose; Thin upper lip; Mild eversion of lower vermillion; High-arched palate; Prominent chin; Facial hair; Small ears	NA	Broad forehead; Small palpebral fissure; Cow’s lick on forehead; Deep and prominent concha	Flat face; Deep set eyes; Hypertelorism; Bulbous nose; Long philtrum; Thin upper lip; Low-set and posteriorly rotated ears; Deep and prominent concha; Broad forehead	Epicanthus	Medial eyebrow flare; Hypertelorism; Wide nasal root; Depressed nasal tip; High broad forehead	cupped ear, long face and short nose	—	10/11
Other symptoms	NA	NA	NA	Hypertrichosis	Three cafe-au-lait spots and one hypochromic macula	Hyperplasia congenital surrenales	NA	Respiratory problems (including chronic bronchitis); Head injury at age of 13 years	NA	NA	Recurrent urinary tract infections; Asthma; Huge gain of weight at 3 years	Cutis marmorata	—	—	

Abbreviations: HP, human phenotype (https://hpo.jax.org/); P, patient; NA, not available; y, years; m, months; EEG, electroencephalography.

The mechanism by which *UBAP2L* variants cause developmental delay, speech delay, mild to severe intellectual disability (ID), seizures, hypotonia, behavioural abnormalities, hypotonia, skeletal and facial abnormalities, and other clinical symptoms remains unclear. There are multiple potential mechanisms by which *UBAP2L* may influence neurodevelopmental disorders. The UBAP2L protein consists of 483 amino acids and includes an N-terminal ubiquitin-associated (UBA) structural domain, which is involved in the ubiquitin-proteasome system and in the formation of aggregates induced by proteasome inhibitors (Maeda et al., 2016; Wilde et al., 2011). *UBAP2L* is a spindle-associated protein that plays a crucial role in cellular mitosis (Guerber et al., 2023). Previous studies have also reported that UBAP2L functions as a sperm protein that interacts with zona pellucida 3 in the human egg, playing an important role in the fertilization process (Naz and Dhandapani, 2010). Additionally, it is involved in the formation of complexes that regulate the activity of hematopoietic stem cells (Bordeleau et al., 2014). Furthermore, UBAP2L is implicated in the formation of stress granules (SGs), which are essential for cell survival under stress conditions and have been linked to the pathogenesis of cancer, neurodegeneration, inflammatory diseases, and viral infections (Panas et al., 2016; Buchan et al., 2008; Arimoto et al., 2008; Takahara and Maeda, 2012). Further functional studies are needed to enhance our understanding of the disorders associated with *UBAP2L* and the mechanisms by which they operate.

Among the identified variants, the *SAMD12* variant (NM_207506.3, c.427C>T (p.Arg143*)) was a nonsense variant, present in the Genome Aggregation Database (gnomAD v.2.1.1) and dbSNP Database (rs764723335) with a minor allele frequency of 0.000007 (Supplementary Table S1). The variant is located in the last exon of the *SAMD12* gene and results in a premature termination codon that produces a truncated protein, however, it remains unclear whether the variant causes nonsense-mediated decay (NMD). Variants in *SAMD12* are associated with benign adult familial myoclonic epilepsy type 1 (BAFME1, OMIM 601068), a rare autosomal dominant disorder characterised by adult-onset cortical myoclonus with or without seizures (Ishiura et al., 2018; Cen et al., 2018; Lei et al., 2019). It has been reported to be associated with intronic TTTCA expansions. The molecular aetiology of the TTTCA repeat expansions in the pathogenesis of BAFME1 has not yet been elucidated. In this study, the proband’s seizure type is absence seizure with high fever. The father, who carries the same variant, has not yet shown any of these symptoms. Taken together, this variant is currently considered to be of uncertain clinical significance and therefore not responsible for the phenotype in the patients. However, as the onset of BAFME1 is usually in adulthood, long-term follow-up of these patients is necessary to assess possible disease progression.

## Conclusion

In conclusion, we report the first familial cases of UBAP2L-deficiency syndrome with a novel loss-of-function variant *UBAP2L* identified in China. The proband in this family presented a clinical phenotype similar to NEDLBF, which includes intellectual disability, developmental delay, speech delay, facial dysmorphism, seizures, and behavioral abnormalities. The affected mother presented only mild intellectual disability and mild language impairment. The phenotypic variability associated with different *UBAP2L* variants suggests that further functional studies on the specific functions of these variants will enhance our understanding of the disease and its underlying mechanisms. The detailed genotype and phenotype information presented in this study is highly significant for the genetic diagnosis and genetic counselling of patients with UBAP2L-deficiency syndrome.

## Data Availability

The datasets presented in this study can be found in online repositories. The names of the repository/repositories and accession number(s) can be found in the article/Supplementary Material.
